# Delivering NICE Joint Pain Advice in the workplace

**DOI:** 10.1002/msc.1539

**Published:** 2021-03-02

**Authors:** Michael V. Hurley, Sally Irwin, Jo Erwin, Amber Gibney, Rachel Hallett, Andrea Carter, Anthony Woolf

**Affiliations:** ^1^ Faculty of Health, Social Care and Education St George's University of London and Kingston University London UK; ^2^ Musculoskeletal Programme Health Innovation Network London UK; ^3^ Bone & Joint Research Group Royal Cornwall Hospitals NHS Trust Truro UK

**Keywords:** back pain, hip, knee, self‐management, workplace

## Abstract

**Background:**

Chronic joint pain is extremely prevalent, but its impact can be mitigated if people receive self‐management/lifestyle advice, especially about the importance of physical activity and maintaining a healthy weight. To reach the large number of people who needs support, we devised Joint Pain Advice (JPA), an intervention that can be delivered in a variety of health and community settings by a range of healthcare and non‐healthcare professionals. Here we extend JPA delivery into workplace settings.

**Method:**

In each workplace, an advisor was trained to deliver JPA. This involved an initial assessment of participant's pain, musculoskeletal health and function (MSK‐HQ), number of days/week active for >30 min, and physical function. Participants were taught simple self‐management strategies, encouraged to adopt healthier lifestyles using motivational interviewing, goal‐settings and personalised action/coping plans. Participants were reviewed three times over 6 months, baseline outcomes reassessed, progress highlighted, health messages reinforced and action plans revised, if necessary.

**Results:**

Twenty large public organisations or small/medium enterprises delivered JPA to 481 people. Satisfaction with the service was high; people found it acceptable, valued advice tailored to their individual needs and experienced tangible benefits—MSK‐HQ (9.5 points; CI 8.3 to 10.6), pain (−1.7; −2.2 to −1.7), physical function (−2.0; −2.2 to −1.7), activity levels and self‐confidence improved, whilst absenteeism and healthcare utilisation reduced.

**Conclusion:**

Delivering advice about self‐management for chronic knee, hip and back pain in workplace settings using local health promotion or occupational health professionals and is practicable, beneficial and valued. JPA could benefit small, medium and large employers.

## 1 INTRODUCTION

Worldwide back pain and chronic knee and hip pain (often labelled osteoarthritis [OA]) are leading causes of pain, disability, reduced mobility, physical and psychosocial health and well‐being and quality of life (Hunter, Schofield, & Callander, 2014; Vos et al., 2020). In the United Kingdom, knee and hip OA affects 9 million people (Versus Arthritis, 2019), and about 10 million people have chronic low back pain (Li, Gignac, & Anis, 2006a; Versus Arthritis, 2019). These problems impact people's personal, social and working lives, impairing physical ability and mental well‐being, causing anxiety, depression and reduced self‐confidence (Versus Arthritis, 2019). They also have a wide socioeconomic impact through substantial health and social care expenditure and lost productivity (Li et al., 2006a). In the workplace, they can force people to change duties, reduce hours, and take sick leave and early retirement (Li et al., 2006a). Each year OA results in 3 million lost working days and back pain 4 million lost working days (Versus Arthritis, 2019) and one in four people who consult their GP about OA leave the workplace prematurely (Conaghan et al., 2015). As the prevalence of joint pain increases with age and the working age is extending, the impact of joint pain will increase (Business in the Community, 2017; National Institute for Health and Clinical Excellence, 2009, 2014; NHS England, 2018; Public Health England, 2017).

International guidelines for the management of OA (National Institute for Health and Clinical Excellence, 2014) and back pain (National Institute for Health and Clinical Excellence, 2009) recommend providing people with information about the causes of their problems, along with reassurance, advice and self‐management strategies, particularly emphasising the importance of physical activity and maintaining a healthy body weight, to improve all areas of people's lives. Chronic joint pain is usually managed by GPs, but employers are not legally required to allow employees time off work to see a GP. Moreover, GPs lack the time needed to enable people to adopt healthier lifestyles, such as supporting increase in physical activity and support weight loss (Basedow & Esterman, 2015; Cottrell, Roddy, Rathod, Porcheret, & Foster, 2016; Hurley, Walsh, Bhavnani, Britten, & Stevenson, 2010; Porcheret, Jordan, & Croft, 2007) or to provide the specific advice necessary to help someone remain in or return to work.

To reach the large number of people needing better advice, we designed a ‘Joint Pain Advice (JPA)’ service enabling a range of healthcare and non‐healthcare professionals to deliver NICE advice. In previous studies, people have found JPA easy to access, convenient, acceptable and effective in helping them become more active, and consequently have experienced less pain and better physical, mental and emotional well‐being. They also report needing fewer medical consultations, investigations, medication and interventions (Hurley, Semple, Sibley, & Walker, 2019; Walker et al., 2017a) with an estimated social return on investment of 1:4 (Walker et al., 2017b).

Delivering JPA in workplaces could provide working populations with easier access to better care that increases their physical and mental health and well‐being, enabling them to carry out their work‐related activities, reducing absenteeism (off work due to ill health [Demou et al., 2018]) and presenteeism (going to work despite illness [Skagen & Collins, 2016]) and creating a healthier, happier workforce. This study evaluated the feasibility of setting up and delivering JPA services in small and medium enterprises (SMEs) and large public organisations.

## 2 METHODS

### 2.1 Design

A service evaluation delivered in workplaces to assess feasibility and effectiveness.

### 2.2 Participants

To take part in the study: *Employers* had to be willing to allow existing employees time to be trained and to deliver JPA to employees, or allow peripatetic Advisors to deliver the service, and allow employees time off work to engage with the service; *Employees* had to have had knee and/or hip and/or back pain for at least 3 months, be over 40 years old experiencing knee or hip pain or over 18 years old if experiencing back pain, and be able to take time away from work to attend appointments; *Advisors* could be existing staff members who may be members of the occupational health department if the organisation was large enough to have one, or a peripatetic Advisor who travelled to several organisations. Advisors had to undertake a half‐day training course that taught them the ethos of JPA, its content, format and delivery, and how to measure the outcomes. The Advisors liaised with the employers and employees to book baseline assessments, deliver JPA and book the review appointments. They also contacted participants who cancelled or did not attend appointments to see why, re‐book or determine reasons for withdrawal as appropriate, and collect feedback on the service.

### 2.3 Intervention

JPA involved employees with chronic hip and/or knee and/or low back pain attending up to four 30‐min face‐to‐face consultations, over 6 months with a trained Advisor (Hurley et al., 2019; Walker et al., 2017a; Table 1). The initial baseline consultation involved the Advisor taking a relevant history of each employee's joint pain and how it affected their working and social life. They conducted ‘clinical’ measures of their pain, musculoskeletal health, physical ability, activity levels and quality of life. The Advisor then worked collaboratively with participants explaining the possible causes of pain, helping participants understand how they could help themselves and provided ‘supported self‐management’.

**TABLE 1:** Outline of the Joint Pain Advice service

Timepoint	Content of review consultation
Initial (baseline) consultation	Assessment of physical function, pain and symptoms, quality of life and lifestyle, number of sit‐to‐stands performed in 30 s, number of days a week physically active for 30 min or more
	Co‐development of an individualised action plan tailored to each person's needs
	Encourage physical activity
	Simple pain management techniques (hot/cold packs; rest/activity cycling)
	Weight reduction, if necessary, to achieve and maintain a healthy body weight
	Signposting to activities in local area to support action plan, for example, exercise and healthy eating
3‐week review	Repeat baseline measures (e.g., sit‐to‐stands, days physically active for 30 min or more a week) and feedback progress
	Reinforce health messages and advice
	Provision of on‐going support, reassurance, motivation and encouragement
6‐week review	Repeat baseline measures and feedback progress
	Revision of goals (if appropriate)
	Reinforcement of health messages and advice
	Provision of on‐going support, reassurance, motivation and encouragement
	Participants encouraged to take up activities through sign‐posting if they have not already done so
6‐month review	As above

Behavioural change techniques such as motivational interviewing, goal setting and action planning and pain coping and self‐management strategies (rest‐activity cycling, use of hot/cold packs, etc.) were used to nurture healthier lifestyles, in particular the importance of being active and maintaining a healthy body weight (Hurley et al., 2019; Walker et al., 2017a). An introduction to motivational interviewing was taught during the training; additional resources were provided and were encouraged to practice MI before starting appointments. Instead of providing ‘answers and solutions’, Advisors elicited from participants their experiences of living with joint pain, aims, ambitions, potential barriers, how these might be overcome and gave participants the major role in deciding what they wanted to do and how to achieve this.

Subsequent review consultations approximately 3 weeks, 6–8 weeks and 6 months later repeated the outcome measures, reviewed progress, fed this back to the employee, reinforced the health messages and provided advice. They gave reassurance, motivation and encouragement to engage with local work or community activities and initiatives, such as walking groups, exercise classes or weight loss programmes where appropriate. This fostered a personalised, collaborative approach that enabled participants time to consider options and make changes alongside collaborative conversations.

The extent to which Advisors could address participant's concerns depended on the scope and boundaries of their existing role, skills and competency. Advisors highlighted accredited online resources and information (NHS, Public Health England, Versus Arthritis, Chartered Society of Physiotherapy, British Heart Foundation, etc), signposted participants to community services and, where necessary, participants were encouraged to speak with a GP, pharmacist, healthcare professional or social worker for health, psychological or social issues raised that were beyond their expertise.

### 2.4 Outcomes

Descriptive data were collected of employee's age, gender, joint(s) affected, employment status and the sedentary/manual nature of their work. In addition, self‐reported absenteeism and healthcare utilisation (medication usage, GP appointments) was taken at baseline for the 6 months prior to taking part in the JPA service and at 6 months for the 6 months of JPA. To evaluate the effectiveness of the JPA service, the quantitative outcomes collected at all appointments were; Musculoskeletal Health Questionnaire (MSK‐HQ; Hill et al., 2016) which assesses people's physical, mental and emotional health and well‐being, ability to work and quality of life related to musculoskeletal problems; pain experienced over the previous 2 weeks on a Numerical Rating Scale of 0–10, where 0 = no pain and 10 = the worst pain they have ever had; Physical ability the number of sit‐to‐stands a participants could perform from a chair without using their arms in 30 s (Jones, Rikli, & Beam, 1999); Physical activity was determined by the number of days per week participants reported they undertook ≥30 min of moderate intensity physical activity; their confidence in their ability to self‐manage their problems. These outcomes were also shared with each employee to feedback progress, reinforce health messages and motivate participants.

Satisfaction with the JPA service was determined by inviting all participants to complete an anonymous online satisfaction questionnaire, and the NHS Friends and Family Test where participants were asked if they were ‘very likely/likely/neither likely nor unlikely/unlikely/very unlikely’ to recommend the JPA service to family and friends.

Referral rates, uptake, ‘failure to attend’ and adverse events were recorded to determine feasibility and acceptability of the service.

### 2.5 Data analysis

To evaluate the effectiveness of the JPA service, the clinical outcomes were summarised using descriptive statistics, and paired two sample *t*‐tests used to compare the means between baseline and review assessments. For healthcare utilisation, self‐reported usage in the 6 months prior to baseline assessment was compared to the healthcare utilisation between baseline and the 6 months assessment. Statistical significance was set at *p* < 0.05. Statistical analysis was conducted in Rstudio (Version 1.1.383; Rstudio Inc.). Satisfaction with and acceptability of the JPA service was assessed from the percentage responses to the satisfaction questionnaire, attendance and retention of employees on the JPA service.

## 3 FINDINGS

Twenty public organisations and private enterprises took part in the study. Employees were recruited by leaflets, posters, emails, intranet bulletins, team meetings, referral from occupational health or self‐referral. We cannot determine uptake of JPA as we do not know if people saw posters, opened emails and so on; how many of those who did had (i) knee, hip and/or back pain, (ii) were eligible and (iii) attended JPA. In total, 481 people (74% female) accessed JPA average age of 49 years, most (39%) were between 45 and 54 years, only a few were over 65 years (Table 2). Despite people having back (32%), knee (21%) and hip (4%) pain or pain at several joints (42%), the majority (97%) continued to work in predominantly sedentary occupations (75%).

**TABLE 2:** Characteristics of participants

Age	*n*	%
18–24	10	2
25–34	48	10
35–44	76	16
45–54	187	39
55–64	142	30
65+	14	3
Total	477	100

Joint affected		
Back	154	32
Knee	100	21
Back, hip and knee	67	14
Back and knee	63	13
Back and hip	40	8
Hip and knee	32	7
Hip	21	4
No pain	6	1
Total	483	100

Employment status		
Working	381	97
On sick leave	12	3
Total	393	100

Nature of work activities		
Sedentary	359	75
Manual/active	119	25
Total	478	100

Abbreviations: *n*, number; %, percentage.

### 3.1 Retention

Overall, JPA attendance and completion rates were moderate, withdrawal usually occurred after the first appointment, retention at 3 weeks was 79%, at 6 weeks 675 and at 6 months 53% (Table 3). Reasons for attrition included joint pain improved/resolved, ineligibility, unable/unwilling to commit time due to work or personal commitments, expecting ‘a quick fix’, expecting a clinical intervention, other health issues, inconvenient appointment times and employers not releasing employees to attend appointments.

**TABLE 3:** Attrition rate from Joint Pain Advice service

		Retention					
	Baseline	3‐week review		6‐week review		6‐month review	
Location	*N*	*N*	%	*N*	%	*n*	%
Total	481	382	79	322	67	256	53

### 3.2 Benefits

Following JPA, there were significant improvements in employee's MSK health (MSK‐HQ), pain, function, disability and physical activity (Table 4).

**TABLE 4:** Changes in outcomes between baseline and subsequent review

	*n*	Baseline	Review	Change (CI)	Effect size
Variable			3 weeks		
MSK‐HQ	381	32.4	38.1	5.8 (5.1 to 6.4)	0.85
Pain Scale^a^	373	5.6	4.5	−1.1^a^ (−1.3 to −0.9)	0.56
Sit‐to‐stands	360	11.5	13.5	2.0 (1.4 to 2.4)	0.41
Days of physical activity	374	2.8	3.7	0.9 (0.7 to 1.1)	0.46
Physical function^a^	374	4.7	3.7	−1.1^a^ (−1.3 to −0.9)	0.54
Work and daily routine^b^	381	2.5	2.8	0.4 (0.3 to 0.4)	0.40
Emotional well‐being^c^	381	2.6	2.9	0.3 (0.2 to 0.4)	0.36

	*n*	Baseline	6 weeks	Change (CI)	Effect size
MSK‐HQ	318	32.8	40.5	7.7 (6.8 to −8.5)	0.99
Pain Scale^a^	315	5.5	3.9	−1.5^a^ (−1.7 to −1.3)	0.73
Sit‐to‐stands	307	11.7	14.5	2.8 (2.2 to 3.4)	0.52
Days of physical activity	316	2.8	3.7	0.9 (0.7 to 1.2)	0.43
Physical function^a^	315	4.6	3.2	−1.4^a^ (−1.7 to −1.2)	0.67
Work and daily routine^b^	318	2.5	3.0	0.4 (0.3 to 0.5)	0.47
Emotional well‐being^c^	318	2.7	3.2	0.5 (0.4 to 0.6)	0.51

	*n*	Baseline	6 months	Change (CI)	Effect size
MSK‐HQ	251	33.4	42.8	9.5 (8.3 to 10.6)	1.04
Pain Scale^a^	254	5.3	3.4	−1.9^a^ (−2.2 to −1.7)	0.85
Sit‐to‐stands	244	11.7	14.8	3.1 (2.3 to 3.9)	0.50
Days of physical activity	256	2.9	3.5	0.7 (0.4 to −1.0)	0.30
Physical function^a^	255	4.6	2.6	−2.0^a^ (−2.2 to −1.7)	0.89
Work and daily routine^b^	251	2.6	3.2	0.6 (0.5 to 0.7)	0.60
Emotional well‐being^c^	251	2.8	3.3	0.5 (0.4 to 0.7)	0.49
Confidence to self‐manage	256	5.5	7.8	2.3 (2.0 to 2.6)	0.85
Work absenteeism/days	247	4.1	2.0	‐	‐
GP consultations	240	1.2	0.5	‐	‐

*Note*: we can be 95% confident that the average change falls within this range. Confidence intervals that do not include 0 is statistically significant at *p* < 0.05. Effect size—the difference between two groups, an effect size of around 0.2 would be considered a ‘small’ probably trivial difference people would not be aware of, an effect size around 0.5 represents a ‘medium’ difference that might affect people's lives, an effect size around 0.8 would be considered a ‘large’ change that people would be aware affects their lives.

Abbreviations: CI, 95% confidence interval.

^a^ Lower scores better; all other variables higher scores better.

^b^ MSK‐HQ Question 6—How has your joint pain affected you work and daily routines?

^c^ MSK‐HQ Question 11—Anxiety and low mood?

### 3.3 Satisfaction

In the ‘Friends and Family’ test 91% of participants said, they would recommend the JPA service to their family and friends. One hundred and fifty‐three of 453 participants (29%) completed an online survey of the JPA service. Most participants (92%) were ‘very satisfied’ with the service, attributed the improvements they experienced and reduced healthcare utilisation to JPA, and said they would recommend the service to family and friends. They valued one‐to‐one, unrushed appointments, felt listened to and thought the advice they received was clear, realistic and practicable, and the action plan was personalised to each participant. As a result, they reported they understood pain better and 79% felt more confident in their ability to manage their pain. People thought delivering JPA in workplace settings was appropriate, convenient and meant they did not have to take time off from work. They felt empowered to request adjustments to their workplace environment and practices, and 62% reported changing the way they did things at work.

### 3.4 Healthcare utilisation

**FIGURE 1:**
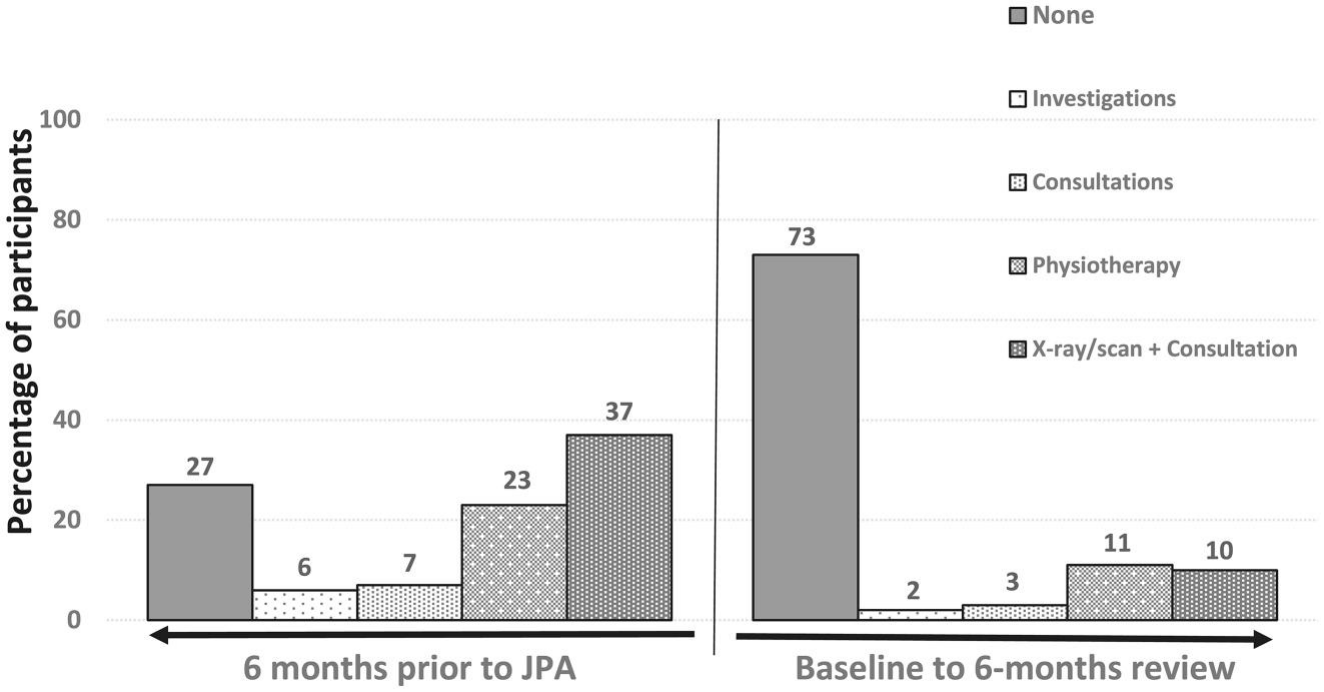
Healthcare utilisation (consultations, investigations and interventions) in the 6 months prior to starting JPA compared to 6 months after JPA. Presented as percentage of participants having a consultation, investigation or intervention

In the 6 months prior to JPA, the majority of participants (291 of 399, 73%) had accessed some form of healthcare—a consultation, investigation and/or intervention (Figure 1) and most (81%) were taking medication for their knee, hip and/or back pain. In the 6 months after initiation of JPA, the majority of participants had no investigations (73%), fewer people had consulted about or had interventions for joint pain (Figure 1; Table 4) and the number of participants taking medication, the number of medications taken and frequency of medication (i.e., taken daily, weekly, monthly or rarely) decreased. Fewer workdays were lost in the 6 months following initiation of JPA compared to the previous 6 months (Table 4).

### 3.5 Costs

Delivering JPA involved salary of Advisors for 4 × 30 min appointments, administration time (∼30 min per participant) and supervision of Advisor and administrator (∼15 min per participant). Based on the 2020 UK NHS pay scales (incl. 23% on‐costs):

Advisor (UK NHS Band 6 entry point) 2 h = £46.76 (€53; $63)

Administrative support (UK NHS Band 4 entry point), 30 min = 8.16 (€9; $11)

Supervision (UK NHS Band 7 entry point), 15 min = 7.25 (€8; $10)

Total = £62.01/employee (€70; $83).

## 4 DISCUSSION

This is the first time work‐based professionals have been trained to deliver JPA to employees with back, knee and/or hip pain, using ‘in‐house’ or peripatetic Advisors. For a relatively modest investment, it is possible to deliver such as service in small, medium and large public organisations and private enterprises. Satisfaction with the service was very high, with improvements in pain, physical, mental and emotional well‐being, and reductions in pain, absenteeism and use of healthcare services.

To implement JPA and maximise recruitment, retention and benefits, employers and employees needed to appreciate the time, effort and resources needed to deliver JPA. ‘Buy‐in’ from employers and line‐managers was vital when planning, promoting, encouraging and ensuring employees had adequate time to engage with JPA (Quirk, Crank, Carter, Leahy, & Copeland, 2018). Employees need to understand what the service offers them and their commitment. Although some individuals came with unrealistic expectations of a ‘quick fix’, the majority appreciated the need for effort and commitment from them. Some had not been properly informed about the JPA service, some attended because they were told to, some thought it was only a one‐off appointment and some were expecting to see a doctor or physiotherapist. These unrealistic misunderstandings may have contributed to unnecessary attrition, which could have been avoided by providing participants with better information about JPA—what it was (not), who it was for and what it entailed.

SMEs account for 99.9% of private businesses (Department for Business Innovation and Skills, 2014) in the United Kingdom, but few have the resources to run occupational health departments. Successfully delivering JPA services in SMEs shows they have the ability to improve joint care and reduce the burden of lost productivity, so like larger organisations with more resources and occupational health departments they also have the ability to look after and improve the musculoskeletal‐related physical and psychosocial health and well‐being pf their employees.

The estimated cost of JPA was relatively modest. For an investment of about £62/employees [€70; $83], employers have employees with better physical and mental well‐being, who take one less sick day in 6 months, attend fewer GP appointment and have fewer investigations and interventions saving time away from work, and fewer medications and possible side‐effects. It is difficult to compare the cost of JPA to similar interventions as nothing similar to JPA has been documented in workplaces. Joint pain is usually managed by GPs (Porcheret et al., 2007) and physiotherapists (Walsh & Hurley, 2005), a 9‐min GP consultation costs £33 [€37; $44] (Curtis, 2019) so four GP consultations would cost £132 [€148; $177]. Physiotherapy (Band 6) costs £23.50 [€26; $32] (Curtis, 2019) for a 30‐min assessment, so four sessions would cost £94 [€106; $126]. Although GPs advise people to lose weight and take exercise this is rarely sufficient to effect behavioural change and get them to adopt healthier lifestyles (Walsh & Hurley, 2005). Physiotherapy aims to reduce pain and improve function, but not sustained behavioural change, self‐management or self‐reliance, so subsequent courses of physiotherapy, GP visits, investigations and interventions are usually necessary. However, accessing these healthcare services is difficult, and following the recent COVID‐19 pandemic likely to be more so. JPA not only improves access to better care it also signposts participants to services offered in their workplace or communities (e.g., exercise, weight management, smoking cessation) to help them adopt healthier lifestyles, which would produce a healthier, happier work force, reducing absenteeism, presenteeism and maximising productivity.

### 4.1 Strengths and limitations

The main strength of this study is its pragmatic design. Our study is a ‘realist service evaluation’ that tells us what will happen when we deliver a complex health intervention (such as ESCAPE‐pain) under ‘real world’ conditions. A number of small, medium and large organisations and enterprises agreed to take part, of whom almost 500 employees chose to take up the service. The benefits and high user‐satisfaction reflect the findings of previous studies (Hurley et al., 2019; Walker et al., 2017a). These factors increase the likelihood that the findings can be replicated in other workplaces, and we have a clear idea of what we must do and avoid doing to maximise resources, time and effort and replicate the benefits attained.

However, the programme's highly pragmatic nature gives rise to limitations that need to be borne in mind when considering the findings. First, participants chose to attend JPA and taking part required an investment of their time and effort, so ‘volunteer bias’ may have made them more likely to exaggerate the self‐reported outcomes. Similarly, the Advisors could have inflated the outcomes they assessed. Rigorously designed research trials are needed to corroborate our findings. However, conducting such trials is extremely difficult and they do not always reflect real‐world contexts which can compromise their generalisability.

Second, there was a steady decline in the number of participants returning for review giving rise to missing data, and we do not know what happened to all of those participants. Participants who Advisors contacted after they failed to attend an appointment often said they felt they had received enough advice in the initial appointments to enable them to self‐manage their pain better themselves, so they no longer needed the service. This is corroborated by the high satisfaction with the JPA service, and matches high withdrawal despite high satisfaction we found in previous studies (Hurley et al., 2019; Walker et al., 2017a). We contend that high withdrawal does not reflect the failure of an ineffective or unneeded service, but rather people self‐managing their problem by choosing what help they needed, when they needed it and which was the prime aim of the JPA service. Creating flexible, accessible, efficient services that can be accessed when needed (Davison, 2000; Maddison et al., 2004; Smink et al., 2011; von Korff & Moore, 2001; Walker et al., 2018) maximises the efficiency of JPA by targeting care to people who need it, when they need it.

Finally, some of the costs of JPA cannot be easily incorporated in the estimated costs of the service [£62; €70; $83]; these include planning, initial and on‐going implementation of the service and loss in productivity incurred releasing employees to attend appointments. Some of these will be one‐off costs that will be offset over time, and staff time is likely to be recouped through reduced absenteeism and presenteeism. Furthermore, the estimated cost only reflects the cost of the ‘in house’ model; the costs of the ‘peripatetic’ model is harder to estimate as it depends on who is delivering the service, and how it is set up. In addition, healthcare utilization, presenteeism and absenteeism were self‐reported, and we did not have the resources to corroborate. However, self‐report has been shown to be reasonably accurate (Beckett, Weinstein, Goldman, & Yu‐Hsuan, 2000) in relatively young people, for a slowly progressive condition such as joint pain, that entails few investigations, interventions and medication.

In summary, in spite of chronic joint pain being very common and a major cause of lost work productivity, (Versus Arthritis, 2019; Conaghan et al., 2015; Business in the Community, 2017; NHS England, 2018; Public Health England, 2017) this is the first study we are aware of that trained work‐based professionals, including non‐healthcare professionals, to help give people easier access to information and advice on how they can self‐manage joint pain. A JPA service can be established not only in large organisations, which have occupational departments, but also in SMEs that comprise practically all private businesses in the United Kingdom. JPA can help employees adopt healthier behaviours and lifestyles that improve physical, mental and socioeconomic well‐being and productivity at work.

There are now many types of ‘champions’, such as health and safety, fire and mental health champions, who are trained to make workplaces safer, healthier environments. People trained to advise and look after the large and growing number of people with chronic knee, hip and/or back pain in the workplace could bring significant returns for relatively little outlay.

## CONFLICT OF INTEREST

None of the authors have any conflicts of interest.

## ETHICS STATEMENT

This study was a service evaluation and was not deemed to require ethical approval.

## AUTHOR CONTRIBUTION

Michael V. Hurley – involved in conception of the study; design; obtaining funding; supervised execution of the study; collection, collation and analysis of the data, led preparation of the manuscript. Data guardian for the study. Sally Irwin – oversaw running of the study on a daily basis; training of Advisors; liaison with sites and Advisors; collection and collation of the data; contributed to drafts final manuscript. Jo Erwin– involved in conception of the study; design; obtaining funding; supervised execution of the study; contributed to drafts of the final manuscript. Amber Gibney – supervised collation and analysis of the data; of the contributed to drafts final manuscript. Rachel Hallett – collation of data; contributed to drafts of the final manuscript. Andrea Carter – involved in conception of the study; design; obtaining funding; co‐supervision of the study; contributed to drafts of the final manuscript. Anthony Woolf – involved in conception of the study; design; obtaining funding; contributed to drafts of the final manuscript.

## Data Availability

Data Availability Statement Data available on request from the corresponding author.
